# A quantitative genome-wide RNAi screen in *C. elegans* for antifungal innate immunity genes

**DOI:** 10.1186/s12915-016-0256-3

**Published:** 2016-04-29

**Authors:** Olivier Zugasti, Nishant Thakur, Jérôme Belougne, Barbara Squiban, C. Léopold Kurz, Julien Soulé, Shizue Omi, Laurent Tichit, Nathalie Pujol, Jonathan J. Ewbank

**Affiliations:** Centre d’Immunologie de Marseille-Luminy, Aix Marseille Université UM2, Inserm, U1104, CNRS UMR7280, 13288 Marseille, France; Institut de Mathématiques de Marseille, Aix Marseille Université, I2M Centrale Marseille, CNRS UMR 7373, 13453 Marseille, France; Present address: Section of Hematology/Oncology, Department of Pediatrics, University of Oklahoma Health Sciences Center, Oklahoma City, OK USA; Present address: Institut de Biologie du Développement de Marseille, CNRS, UMR6216, Case 907, Marseille, France; Present address: Institut de Genomique Fonctionnelle, 141, rue de la Cardonille, 34094 Montpellier Cedex 05, France

**Keywords:** Fungal pathogen, Functional genomics, High-throughput screening, Signal transduction, Networks, Osmotic stress, Epistasis, Bioinformatics, Databases, Mitochondrial unfolded protein response

## Abstract

**Background:**

*Caenorhabditis elegans* has emerged over the last decade as a useful model for the study of innate immunity. Its infection with the pathogenic fungus *Drechmeria coniospora* leads to the rapid up-regulation in the epidermis of genes encoding antimicrobial peptides. The molecular basis of antimicrobial peptide gene regulation has been previously characterized through forward genetic screens. Reverse genetics, based on RNAi, provide a complementary approach to dissect the worm’s immune defenses.

**Results:**

We report here the full results of a quantitative whole-genome RNAi screen in *C. elegans* for genes involved in regulating antimicrobial peptide gene expression. The results will be a valuable resource for those contemplating similar RNAi-based screens and also reveal the limitations of such an approach. We present several strategies, including a comprehensive class clustering method, to overcome these limitations and which allowed us to characterize the different steps of the interaction between *C. elegans* and the fungus *D. coniospora*, leading to a complete description of the MAPK pathway central to innate immunity in *C. elegans*. The results further revealed a cross-tissue signaling, triggered by mitochondrial dysfunction in the intestine, that suppresses antimicrobial peptide gene expression in the nematode epidermis.

**Conclusions:**

Overall, our results provide an unprecedented system’s level insight into the regulation of *C. elegans* innate immunity. They represent a significant contribution to our understanding of host defenses and will lead to a better comprehension of the function and evolution of animal innate immunity.

**Electronic supplementary material:**

The online version of this article (doi:10.1186/s12915-016-0256-3) contains supplementary material, which is available to authorized users.

## Background

Infection of *Caenorhabditis elegans* by its natural fungal pathogen *Drechmeria coniospora* provokes an innate immune response characterized by the expression of antimicrobial peptide (AMP) genes in the worm epidermis [1]. We have focused our attention on the regulation of one group of six AMP genes of the “Neuropeptide-Like Protein” class, *nlp-27*–*nlp-31* and *nlp-34*, found together in a short genomic interval of less than 12 kb [2], which we call the “*nlp-29* cluster”, after the best-studied member of the family. Many genes that play an essential role in controlling *nlp-29* AMP gene expression have been defined, acting together in a relatively complex genetic network. Central to this regulation is a conserved p38 MAPK cascade [3], also required for resistance to intestinal bacterial pathogens [4]. Loss of function of any one of the many genes involved provokes a “No Induction of Peptide after *Drechmeria* Infection” (Nipi) phenotype. After small- and large-scale genetic screens for Nipi mutants [3, 5], our knowledge of anti-fungal innate immunity in *C. elegans* remains, however, fragmentary. Not only are there missing elements from the associated signal transduction pathways, but how these pathways cross-talk with each other and with the mechanisms involved in general homeostatic regulation is currently unclear [4]. Another largely unexplored aspect of the worm’s antifungal innate defenses relates to the potential for cross-tissue communication. We have demonstrated that a second family of AMP genes, called caenacins (*cnc*), including *cnc-2*, are controlled by a cell non-autonomous signal transduction pathway, wherein the nematode TGF-ß, DBL-1, produced in neurons, modulates *cnc-2* expression in the epidermis following *D. coniospora* infection. This pathway does not, however, influence *nlp-29* expression [6], which up until now has been found to be controlled cell-autonomously in the epidermis [3, 5, 7–9]. On the other hand, in *C. elegans*, the disruption of cellular homeostasis in one tissue can influence a stress response in a distant tissue (reviewed in [10–12]); whether this is also the case for *nlp-29* remains an open question.

To address these lacunae, since *C. elegans* lends itself to large-scale functional genomic analyses [13, 14], we undertook a genome-wide RNAi screen for genes involved in the regulation of the AMP gene *nlp-29*, with a well-characterized reporter gene system used in our previous studies [3]. Many pathogens can infect *C. elegans* when cultured in liquid in 96 or 384-well plates (reviewed in [15–17]). Since *D. coniospora* cannot infect worms in liquid, however, we developed a novel solid-based high-throughput assay, using the COPAS Biosort [18] to obtain a quantitative measure of reporter gene expression [19]. In a previous report, we focused on the large family of worm G-protein coupled receptor (GPCR) genes and defined a key role for DCAR-1 that acts as a “damage-associated molecular pattern” receptor, acting upstream of the p38 MAPK cascade [20]. This clearly validated the experimental approach and illustrated the utility of this large-scale reverse genetic screen for identifying individual genes.

Here, we present the full results of the screen, which led to the identification of more than 250 candidate genes. Perhaps surprisingly for such a well-studied organism, there is a relative paucity of functional information available for nematode genes, which stands as a barrier to the interpretation of large-scale studies in *C. elegans*. For example, in the recent WormBase release (WS250), only a quarter of protein-coding genes (5162/20,362) are associated with a concise description, a similar proportion (27 %) has a UniprotKB gene ontology (GO) annotation, and 57 % of them (11,970/20,362) have any type of GO annotation in WormBase. We have therefore attempted to couple several broad *in silico* analytical methods with targeted secondary screening to define groups of genes that potentially act together. In doing so, we have been able to identify several distinct biological processes that play an important role in the antifungal response and obtain, for the first time, a comprehensive view of the regulation of AMP gene expression.

## Results

### A quantitative genome-wide RNAi screen for innate immunity genes

**Fig. 1:**
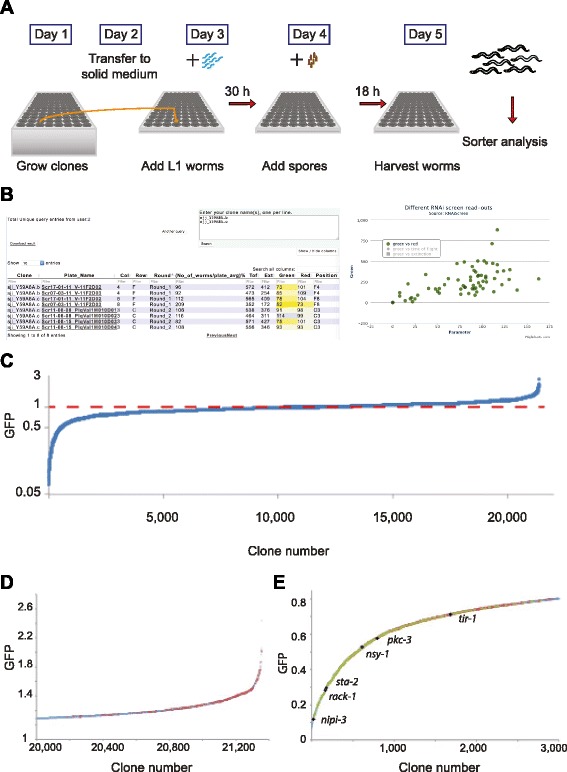
A quantitative genome-wide screen for regulators of AMP gene expression. **a** Simplified overview of the RNAi screen protocol, adapted from [19]. **b** Screenshots from the RNAi screen web interface. Left panel: example of results for two clones (insert at top right: the query box) that target the gene *gck-3*. Contrary to clone sjj_Y59A8A.c that passed the first round of duplicate screening, and for which the results of the second (quadruplicate) round are also displayed, sjj_Y59A8A.b only provoked a 15 % reduction in normalized GFP expression in one of the two first-round tests and so was not retained for the second round. The results for each test are linked to the primary data, which is displayed in the right panel for a single experiment. Users have the option of plotting GFP fluorescence against any or all of three parameters; shown here is GFP versus dsRed expression (in arbitrary units). **c** The ranked averages of the two values for normalized GFP expression for each of the 21,355 RNAi clones tested (21,223 unique clones, 132 present in duplicate), on a log scale. **d** The averages, on a linear scale, of the two values for normalized GFP expression for the last 1,355 RNAi clones. The 295 clones that were retested in a second round are indicated in red. **e** The averages, on a linear scale, of the two values for normalized GFP expression for the first 3000 RNAi clones. The 966 and 360 clones that passed first and second round screening are indicated in red and green, respectively. The results for selected known signaling components are indicated in black

To identify, in an unbiased way, genes potentially involved in the regulation of the induction of antimicrobial peptide genes, we conducted a genome-wide RNAi screen. We first assembled a non-redundant collection of 21,223 RNAi clones from the Ahringer [21] and Vidal [22] libraries (Additional file 1: Table S1). Together, these clones are predicted to target 85 % of the protein coding genes in *C. elegans*. Using an automated method [19], we screened this library twice and quantified the infection-induced expression of the *nlp-29p::gfp* reporter gene in young adult worms (Fig. 1a). The entire set of results, a total of more than 46.8 million data points, including measures of body length (time of flight; TOF), optical density, and reporter gene expression (green (GFP) and red (dsRed)) from the analysis of more than 3.9 million individual worms, is publicly available and can be queried via a dedicated web interface (http://bioinformatics.lif.univ-mrs.fr/RNAiScreen; Fig. 1b). The overall continuous, but far from normal, distribution of the results from the first round of screening (Fig. 1c) is very much in line with previous quantitative large-scale screens in other organisms [23].

### Identification of clones that provoke an exaggerated response

Innate immune responses are limited by negative regulators that contribute to protecting hosts from the collateral damage of their own effector mechanisms [24, 25]. There is emerging evidence that excess NLP-29 can damage host tissue (Dong Yan, Duke University, personal communication). With the aim of identifying negative regulators of the response, in a first step, we retained 295 clones that provoked an average increase of *nlp-29p::gfp* expression of 30 % or more, but that either did not increase the expression of a control transgene, the constitutive epidermal reporter *col-12p::dsRed*, nor the average size of the worms, or if they did, the increase was less than 30 % (Additional file 2: Table S2; Fig. 1d). Inactivation of numerous genes that affect molting, such as *pan-1* [26], the integrity of the cuticle, including *dpy-9*, *osm-11* [2], and *acs-3* [27], or fatty acid metabolism (e.g., *fasn-1*), is known to provoke the “peptide expression no infection”, or Peni phenotype: an elevation of *nlp-29p::gfp* expression in the absence of infection [8]. This is associated with an exaggeratedly high expression after infection too [8], which we call here the Hipi phenotype (for hyper-induction of peptide expression after infection). To identify clones that caused only a Hipi phenotype, the 295 clones were retested in quadruplicate for their effect on *nlp-29p::gfp* expression, both with and without infection. Using cut-offs that captured all the positive controls (*fasn-1* and *pan-1*) but none of the negative controls (*sta-1* and *K04G11.4* [7]), we removed 21 clones that robustly caused a Peni phenotype (Additional file 3: Table S3). Their characterization will be the subject of a future study.

**Fig. 2:**
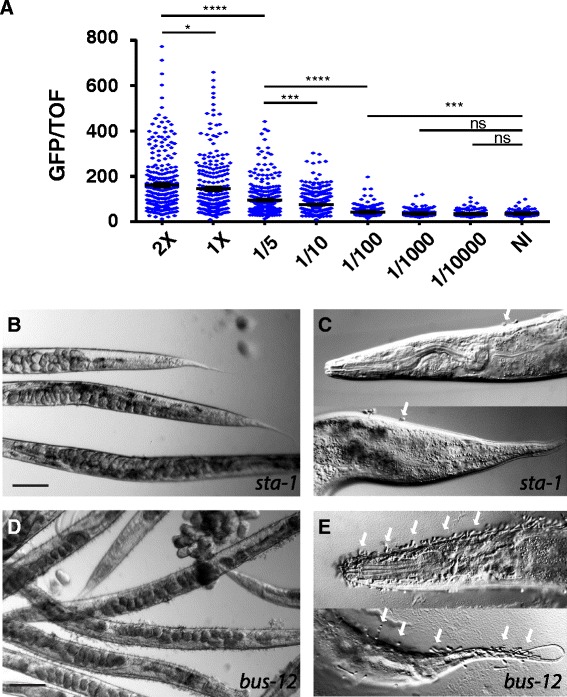
Infection burden affects the strength of the innate immune response. **a** Normalized fluorescence ratio for worms infected for 18 h with the indicated dilutions of a solution of fresh *D. coniospora* spores, compared to non-infected (NI) worms. In each sample, a minimum of 230 worms was analyzed. The bar indicates the mean value. Since spore virulence depends on the age of the spores and of the plate from which they were harvested [121], the absolute spore concentration is not an informative measure and is not shown here. Comparisons between selected conditions are shown (Mann–Whitney test); ns, not significant; * *P* < 0.05; *** *P* < 0.001; **** *P* < 0.0001. **b**–**e**. Comparison at lower (**b**, **d**) and higher (**c**, **e**) magnification between worms treated with a control RNAi (*sta-1*; **b**, **c**) or RNAi against *bus-12* (**d**, **e**). In contrast to the control worms, *bus-12*(RNAi) animals exhibited a very markedly increased adhesion of spores (white arrows) over the entire body (**c**), prominently at the head and tail (**e**). Scale bar in b and d: 50 μm

We then used a simple cut-off to classify 28 clones as being capable of causing a strong Hipi phenotype (termed, “Hipi clones”; Additional file 3: Table S3). We used sequencing and Clone Mapper [28] to verify the identity of the Hipi clones and determine their putative target genes (Additional file 3: Table S3). These included *bus-2* and *bus-12*, which respectively encode a galactosyltransferase and a sugar transporter required for the post-translational modification of surface-exposed proteins [29, 30]. In a detailed analysis, we previously demonstrated that abrogating *bus-2* or *bus-12* function increases spore binding to the nematode cuticle [31]. To address the question of whether the infectious burden of spores affected the strength of reporter gene expression, we exposed wild-type worms carrying *nlp-29p::gfp* to varying doses of *D. coniospora* spores. There was a clear relationship between the concentration of spores and the level of GFP expression (Fig. 2a).

We therefore conducted a third round of screening, this time directly assessing the adhesion of spores to worms treated with the 28 candidate Hipi clones and, in parallel, the degree of expression of *nlp-29p::gfp* relative to worms treated with a control RNAi clone targeting *sta-1*. Half of the clones were again scored as provoking a Hipi phenotype and in each case this was associated with a clear increase in spore binding (Additional file 3: Table S3, Fig. 2b–e). Among the predicted targets of these 14 clones, in addition to *bus-2* and *bus-12*, three other genes are putatively involved in the modification of surface glycans (Table 1). The clone mv_Y38C1AB.5 potentially targets two paralogous genes encoding glycosyltransferases, and is also likely to affect the properties of the cuticle via an effect on surface glycoprotein biosynthesis. Similarly, another predicted target gene, *K08E3.5*, encodes a uridine triphosphate-glucose-1-phosphate uridylyltransferase, expected to be involved in glycoprotein and glycolipid synthesis. While the connection between spore binding and the remaining target genes is less evident and will require further investigation, these results advance our understanding of the interaction between fungal spores and nematode cuticle and emphasize the cardinal importance of the infection burden in determining the strength of the innate immune response.

**Table 1:** Targets of robust Hipi clones

Gene/sequence name	Brief description
*bus-2*	Core-1 beta1,3 galactosyltransferase^b^
*bus-12*	Nucleotide-sugar transporter^b^
*cpt-6*	Carnitine palmitoyltransferase
*sdc-2*	Nematode-specific; required for dosage compensation
*snf-9*	Solute carrier family 6 (SLC6)
*tkt-1*	Transketolase
*ykt-6*	v-SNARE
C14H10.3	Pyridoxal-dependent decarboxylase
K08E3.5	Uridine triphosphate:glucose-1-phosphate uridylyltransferase^b^
F35H12.5	Epoxide/serine hydrolase
K06A9.1	Nematode-specific; limited similarity to mucin
T04G9.4	Aminoadipate-semialdehyde dehydrogenase-phosphopantetheinyl transferase
Y38C1AB.1^a^	Core 1 synthase, glycoprotein-N-acetylgalactosamine 3-beta-galactosyltransferase^b^
Y38C1AB.5^a^	Core 1 synthase, glycoprotein-N-acetylgalactosamine 3-beta-galactosyltransferase^b^
C26B9.3	Nematode-specific

^a^Targeted by a single RNAi clone

^b^Involved in carbohydrate metabolism

### Identification of clones that abrogate the response

To identify positive regulators of the response, following the first round of screening, we retained clones that reduced the infection-induced expression of *nlp-29p::gfp* by 20 % or more in both of the tests (i.e., provoked a Nipi phenotype), but excluded those that altered the expression of the control *col-12p::dsRed* transgene or reduced the average size of the worms more than when we knocked-down the known signaling component *rack-1* [9]. The selected 966 Nipi clones were then tested in quadruplicate, and 360 clones giving a robust Nipi phenotype (Additional file 4: Supplementary Methods) were chosen for further study (Fig. 1e and Additional file 5: Table S5; full results available at http://bioinformatics.lif.univ-mrs.fr/RNAiScreen). Among them, 314 clones were predicted to target a single gene, 22 to target two genes each, two to target three genes each, and eight clones an average of 10 each (due to targeting of multigene families of very similar sequence, e.g., the *his* histone genes; see below). For the remaining 14 clones, no target could be defined because of sequence ambiguity (Additional file 5: Table S5). A total of 404 genes were thus identified as potential targets for these clones, with 15 genes, not counting the many *his* genes, satisfyingly, being hit by more than one clone. The complete RNAi library contained a further 155 clones that potentially hit the same 404 targets (Additional file 5: Table S5). If the clones we have identified are true positives, then these 155 clones should also have been retained. This gives a measure of the efficiency of the screen (360/(360 + 155), 70 %); in common with other RNAi screens (e.g., [32, 33]), false negatives are thus a notable limitation here. Among the identified targets, in addition to *rack-1*, we noted the presence of *nipi-3*, *nsy-1*, *pkc-3*, *sta-2* and *tir-1*, all previously characterized for their role in regulating antimicrobial peptide gene expression [1, 3, 7, 9]. Further, among the clones, we identified one targeting *dcar-1*, encoding a GPCR that we demonstrated to be required for the induction of the innate immune response upon infection [20]. We consider this to be a validation of the screening and selection method.

Going from a genome-wide approach to a more focused analysis allowed attention to be paid to the phenotypes of the worms that had been treated with each of the 360 RNAi clones. A number of clones provoked developmental delays and/or lethality under our experimental protocol. We used the quantitative data from the second round of screening to identify 63 clones associated with pronounced phenotypes (see Additional file 6: Table S4 for criteria). Of these 63 clones, 42 had been associated with severe developmental phenotypes in previous RNAi studies (Additional file 6: Table S4). While we cannot formally exclude the possibility that they exercise both an essential developmental role and a specific role in regulating innate immune defenses, because of their pleiotropic effects they were not included in subsequent analyses. This left us with a list of 297 clones, predicted to target 338 genes (Additional file 5: Table S5), including all the previously characterized genes mentioned above. This list contains many potentially interesting genes, such as *akir-1*, which encodes the worm ortholog of Akirin, a known regulator of innate immunity in flies and mammals [34, 35], the claudin/calcium channel gamma subunit family gene *nsy-4*, known to act upstream of *nsy-1* during neuronal development [36], as well as several genes encoding transcription factors.

Inspection of this list also revealed a potential confounding factor for subsequent analyses. The prediction of targets for an RNAi clone is based on sequence. Several *C. elegans* gene families contain multiple members with highly similar nucleotide sequences, so that a single RNAi clone can have many potential targets. In addition to the clone sjj_K07F5.1, predicted to hit 15 *msp* genes, this principally concerned clones targeting histone genes; a total of 45 *his* genes were identified as potential targets for just eight RNAi clones (Additional file 5: Table S5). In the absence of functional analyses at the single gene level, it is not possible to ascribe the effect of a given RNAi clone to one or multiple targets. Many published genome-wide RNAi screens in *C. elegans* have reported target genes but not RNAi clones, and therefore potentially suffer from this confounding factor that can lead to biases in analyses. For some of our subsequent analyses, we removed these two gene classes, giving a set of 288 clones potentially targeting 278 (Nipi non-*his* non-*msp*) genes (Tables 2 and 3).

**Table 2:** Overview of screen results

Step	Type of clone	Number of clones	Source
Primary screen	Library	21,223	Additional file 1: Table S1
Secondary screen for increased reporter gene expression	Candidate	295	Additional file 2: Table S2
	Peni	21	Additional file 3: Table S3
	Candidate Hipi	28	Additional file 3: Table S3
Tertiary screen for increased reporter gene expression	Hipi	14	Additional file 3: Table S3
Secondary screen for decreased reporter gene expression	Candidate Nipi	966	http://bioinformatics.lif.univ-mrs.fr/RNAiScreen
Tertiary screen for decreased reporter gene expression	Nipi	360	Additional file 6: Table S4
With pronounced developmental phenotype		63	Additional file 6: Table S4
Retained	Nipi	297	Additional file 5: Table S5
Clones targeting *his* or *msp* genes		9	Additional file 5: Table S5
Remainder	Nipi -*his* -*msp*	288	Additional file 5: Table S5

**Table 3:** Enrichment of functional classes among 278 Nipi genes

Category/Phenotype upon RNAi^a^	Number over-lapping genes	Total number genes in class	Percentage of MAPK signaling genes in class	Probability^b^
1. Conserved in *D. melanogaster* ^c^ as described in [124]	169	3710	84.8	3.77 × 10^−54^
2. Stimulate microbial aversion behavior^c^ [26]	65	374	33.3	1.39 × 10^−50^
3. Suppress over-expression of *gpdh-1*p::*gfp* seen in *osm-8* mutant^c,d^ [72]	52	252	24.2	7.87 × 10^−44^
4. Decrease *acdh-1p::gfp* expression [73]	35	146	6.1	3.33 × 10^−31^
5. Induce *hsp-6p::gfp* (mitochondrial UPR) [66]	30	95	0.0	1.60 × 10^−30^
6. Protein expression [125]	43	446	9.1	1.18 × 10^−21^
7. Required for cytoprotective response – four different reporter genes^c^ [49]	21	71	24.2	3.08 × 10^−20^
8. Required for transgene silencing [57]	50	823	24.2	1.25 × 10^−16^
10. Alter RAB-11 sub-cellular localization and transport of the apical membrane protein PEPT-1 [79]	34	426	15.2	3.40 × 10^−14^
11. Synthetic phenotype with *lin-35* [126]	27	252	6.1	3.95 × 10^−14^
12. Required for mitochondrial surveillance and response [74]	14	45	9.1	2.08 × 10^−13^
13. Regulators of *gpdh-1* expression^d^ [75]	18	123	6.1	2.45 × 10^−11^
15. Aberrant GFP::PGL-1 phenotypes [76]	20	170	12.1	6.87 × 10^−11^
16. Suppressors of polyglutamine aggregation [77]	19	173	9.1	9.65 × 10^−10^
17. Mitochondrial [125]	17	160	0.0	2.35 × 10^−8^
18. Upregulated after 12 h of dauer recovery [127]	44	1175	12.1	3.31 × 10^−7^
20. Altered expression in *zpf-1* mutant [128]	21	333	3.0	2.24 × 10^−6^
21. Down regulated in an *ogt-1* mutant [129]	30	670	9.1	4.02 × 10^−6^
22. Altered expression in *rde-4* mutant [128]	17	232	6.1	7.17 × 10^−6^
23. Induce *hsp-70p::gfp* [130]	9	52	9.1	1.03 × 10^−5^
24. Genes connected to miRNA function [131, 132]	8	42	9.1	2.79 × 10^−5^
25. Increase longevity [133]	9	66	3.0	8.78 × 10^−5^
26. Induced after 24 h of *D. coniospora* infection (cDNA microarrays) [2]	21	419	12.1	1.12 × 10^−4^
27. Confer hypoxia-resistance [134]	14	198	9.1	1.98 × 10^−4^
29. Induce *irg-1*p::*gfp* [59]	10	102	0.0	4.37 × 10^−4^
31. Energy generation [125]	10	104	3.0	5.23 × 10^−4^

^a^The numbers refer to the order of the classes in the complete analysis; some redundant or similar classes have been removed (see Additional file 7: Table S6 for complete data)

^b^Bonferroni-corrected Fischer exact score

^c^Class significantly enriched in the group of 33 “non-*his* non-*msp* MAPK pathway genes”

^d^Class related to osmotic stress response

### The central role of MAPK signaling

The main signaling pathway known to regulate *nlp-*29 expression has at its core a conserved p38 MAPK cassette involving *tir-1*, *nsy-1*, *sek-1*, and *pmk-1* [2, 3]. The identification in the screen of *tir-1* and *nsy-1* was an important validation of the approach. The failure to identify *sek-1* represents a clear example of a false negative arising because of our deliberate selection strategy. The corresponding clone (sjj_R03G5.2) did not pass the first round of screening since it only abrogated reporter gene expression in one of the two trials. For *pmk-1*, as the corresponding clone (mv_B0218.3) surprisingly did not provoke a Nipi phenotype, we sequenced it. In common with the 54/388 candidate clones that we sequenced (4/28 Hipi and 50/360 Nipi clones, respectively; Additional file 3: Tables S3 and Additional file 5: Table S5), in our cherry-picked library, the clone annotated as mv_B0218.3 did not contain the expected insert. We returned to our original copy of the Vidal RNAi collection; the corresponding clone there was also incorrect. This is another of the known drawbacks in large-scale RNAi screens: the impossibility of being certain of the identity of every clone.

MAPK signaling is central to innate immune defense in many species, including *C. elegans* and vertebrates [25, 37]. Catalogs of proteins and genes involved in the regulation of MAPK pathways are available for yeast [38], flies [39], and human [40]. Using the Drosophila RNAi Screening Center (DRSC) Integrative Ortholog Prediction Tool [41], we compared the worm orthologs of the members of these lists with our hits. More than 1/5 of the candidates (76/338 Nipi genes; 22 %) had previously been associated with MAPK signaling in at least one other species. The constitution of the list of potential components of MAPK signaling was skewed by the inclusion of multiple histone genes (43/76; Additional file 5: Table S5). Nevertheless, the identification of 33/278 (non-*his* non-*msp*) MAPK-related genes (Table 4) reinforces the idea that MAPK signaling is central to the regulation of AMP gene expression in *C. elegans* epidermis and underscores the conserved nature of this core signaling process.

**Table 4:** Nipi genes linked to MAPK signaling

Gene/sequence name	Brief description
*ccf-1*	Subunit 7 of CCR4-NOT transcription complex
*cct-3*	Gamma subunit of eukaryotic cytosolic (‘T complex’) chaperonin
*cic-1*	Cyclin C
*cyl-1*	Cyclin L
*dic-1*	DEAD/H BOX 26; mitochondrial
*dnc-1*	Dynactin complex subunit p150; DNC-1 is located at cortical microtubule attachment sites
*ego-2*	Bro1 domain-containing protein; positive regulator Notch signaling
*exos-9*	Exosome component 9
*ftt-2*	14-3-3 protein
*hda-1*	Histone deacetylase 1
*ima-3*	Importin alpha nuclear transport factor
*kin-20*	Casein kinase
*let-92*	Catalytic subunit of PP2A (protein phosphatase 2A)
*nap-1*	NAP (Nucleosome Assembly Protein) family
*npp-1*	Nucleoporin
*npp-10*	Nucleoporin
*ogdh-1*	2-oxoglutarate dehydrogenase, mitochondrial
*puf-9*	PUMILIO RNA-binding protein
*pyp-1*	Inorganic pyrophosphatase; predicted to participate in nucleosome remodeling
*rack-1*	Receptor for Activated C Kinase; homolog of G beta [9]
*rbpl-1*	E3 ubiquitin-protein ligase RBBP6 (Retinoblastoma binding protein 6)
*rps-26*	Small ribosomal subunit S26
*tba-2*	Alpha-tubulin
*tba-4*	Alpha-tubulin
*unc-37*	Gro/TLE (Groucho/transducin-like enhancer)
*vhp-1*	MAP kinase phosphatase
*wnk-1*	With no lysine kinase [8]
*xpo-2*	Importin-beta
F19F109	U4/U6U5 tri-snRNP-associated protein 1
K12H44	Signal peptidase complex subunit 3
R1863	Signal recognition particle receptor subunit beta
F20D122	Germinal-center associated nuclear protein; required for mRNA export

References are given for genes previously connected to the regulation of *nlp-29* expression. Each gene is targeted by a different RNAi clone

**Fig. 3:**
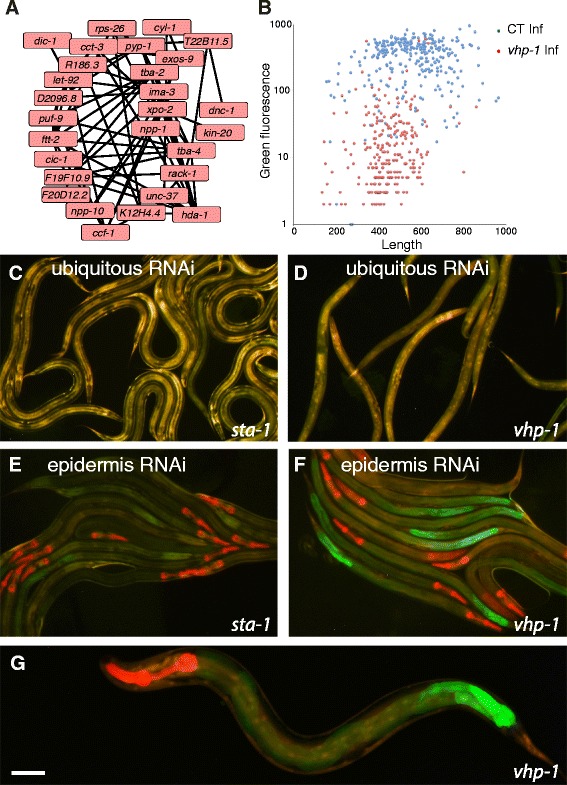
MAPK pathway genes involved in regulation of AMP expression. **a** Interaction network predicted by WormNet for 33 MAPK pathway-related Nipi genes (Additional file 7: Table S6). The genes *ego-2*, *tag-214*, *vhp-1*, and *wnk-1* are not connected to any other of the genes; Y73B3A.18 is not included in the WormNet set of genes. These five genes are not shown here. The remaining 28 genes are connected by 77 edges. As for all large-scale data mining, there are obvious omissions, due to incomplete coverage in databases. One example is *nsy-1* that encodes a MAP3K [122] but does not appear here. **b** Knocking down *vhp-1* by RNAi provokes a marked reduction of *nlp-29p::gfp* reporter gene expression in IG274 worms carrying the *frIs7* transgene infected by *D. coniospora*. The graph shows the quantification of fluorescence of worms treated with control (CT: K04G11.4; blue; *n* = 296) or *vhp-1* (red; *n* = 258) RNAi. The green fluorescence and length are plotted in arbitrary, but constant units. **c**–**g** A cell-non-autonomous regulatory role for *vhp-1*. Whereas following systemic knock-down of *sta-1* (**c**) or *vhp-1* (**d**), there was no detectable expression of the *nlp-29p::gfp* reporter gene in IG274 worms carrying the *frIs7* transgene in the absence of infection, knocking-down *vhp-1* (**f**) but not *sta-1* (**e**) specifically in the epidermis in strain IG1502 led to ectopic expression of GFP in the nematode intestine. This expression was most pronounced in the posterior intestinal cells (**g**). The red fluorescence in the pharynx in (**e–g**) reflects the presence of an additional transgenic marker. Worms were observed at the L4 stage in all cases. Green and red fluorescence are visualized simultaneously with a long pass GFP filter. Scale bar: 50 μm

To explore the functional relationship of the non-histone candidate genes potentially involved in MAPK signaling, we submitted them to an analysis using WormNet, a phenotype-centric tool that represents known interactions between genes in a list [42]. Of the 33 genes entered, 28 formed a well-connected network (Fig. 3a). On the basis of an input list of genes, WormNet predicts other genes that could be functionally related to them, ranked by probability. Among the top 200 WormNet candidates, there were 53 genes that were found as candidate Nipi genes in our screen but that had not been included in the original query of 33 genes. At first sight, this remarkable enrichment would appear to be a testament to the predictive power of WormNet. Inspection of the results, however, showed that 38 of these 53 genes encode histones (Additional file 7: Table S6), which generally share functional annotations. Nevertheless, 15 non-histone genes that had been found in our screen were identified as potentially linked to the MAPK network (Additional file 7: Table S6), suggesting that other WormNet candidates could also be involved in the regulation of *nlp-29p::gfp* expression. Further, this analysis underlines the idea that the genes involved in modulating MAPK signaling are embedded within a broader cellular signaling network.

One of the five MAPK-related candidate target genes that was not part of the MAPK network predicted by WormNet (Fig. 3a) was *vhp-1*, which encodes a member of the VH1 dual-specificity phosphatase family. Since *vhp-*1 has been described as a negative regulator of the p38 pathway [43], we would not have expected to have found it as a Nipi gene. The effect of *vhp-1*(RNAi) on *nlp-29p::gfp* expression after *D. coniospora* infection was very pronounced (Fig. 3b). To determine whether this effect was cell-autonomous, we made use of an epidermis-specific RNAi strain, IG1502 [20]. To our surprise, *vhp-1*(RNAi) provoked a substantial ectopic expression of *gfp* in the intestine in this strain, even in the absence of infection (Fig. 3c–f). The intestine of *C. elegans* is functionally regionalized [44]; *vhp-1*(RNAi)-induced *nlp-29p::gfp* expression was strongest in the posterior intestinal cells (Fig. 3g). Thus, reducing the activity, specifically in the epidermis, of a phosphatase previously shown to down-regulate p38 MAPK signaling leads to ectopic gene expression of a p38 MAPK target in a distant tissue.

### Global functional analyses

**Fig. 4:**
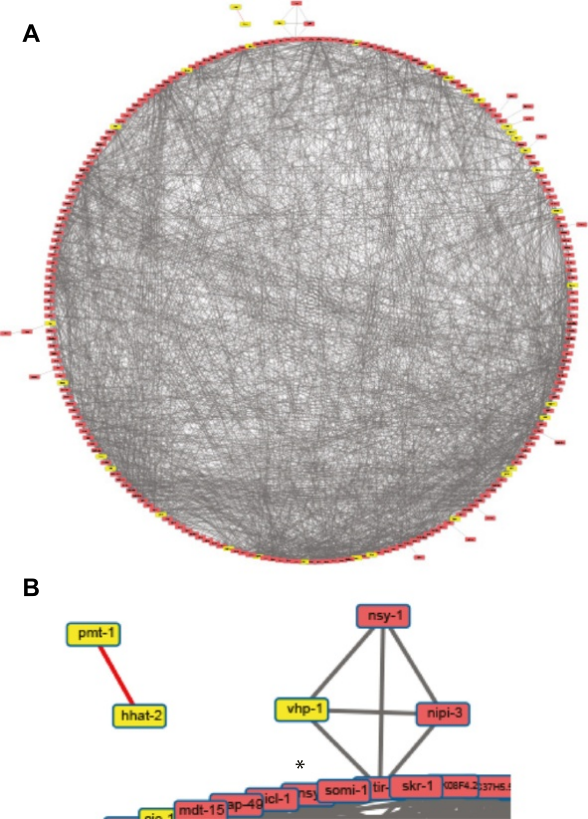
Nipi genes are connected by a dense network of interactions. **a** Interaction network predicted by WormNet for 233 Nipi genes, with 33 MAPK pathway-related genes shown in yellow. **b** Close-up view of one part of the network shown in b, highlighting two interconnected genes that are apart from the main network and illustrating the relative lack of connections for the known signaling components *nsy-1* and *nipi-3*, which are connected to *tir-1*. The gene *nsy-4* is partially obscured; its position is indicated with an asterisk

Returning to a more global analysis, when we submitted the list of Nipi genes (except *his* and *msp* genes) to a WormNet analysis, 231 formed an intensely interconnected network with an average of 11.1 edges/node (Fig. 4). To characterize this broader cellular signaling network, we ran the lists of candidate targets through an Expression Analysis Systematic Explorer (EASE) analysis [45], using our in-house database of functional annotations [46]. A number of classes were significantly enriched (*P* < 10^–3^; Additional file 7: Table S6). Several were derived from early genome-wide ChIP-seq studies produced by the model organism encyclopedia of DNA elements (modENCODE) consortium. We did not exploit this data further since its reliability has recently been questioned by the consortium itself [47].

**Fig. 5:**
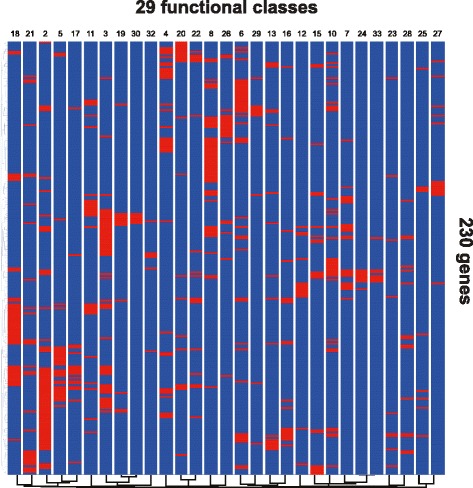
Relationship between different functional classes. Hierarchical clustering of genes and functional classes (see Table 3 for class labels; full data in Additional file 7: Table S6; 15 genes from the 245 candidates, present only in one or more of the classes 1, 9, 14, and 31 are not shown). The presence of a gene in a class is represented by a red rectangle, its absence in blue

**Fig. 6:**
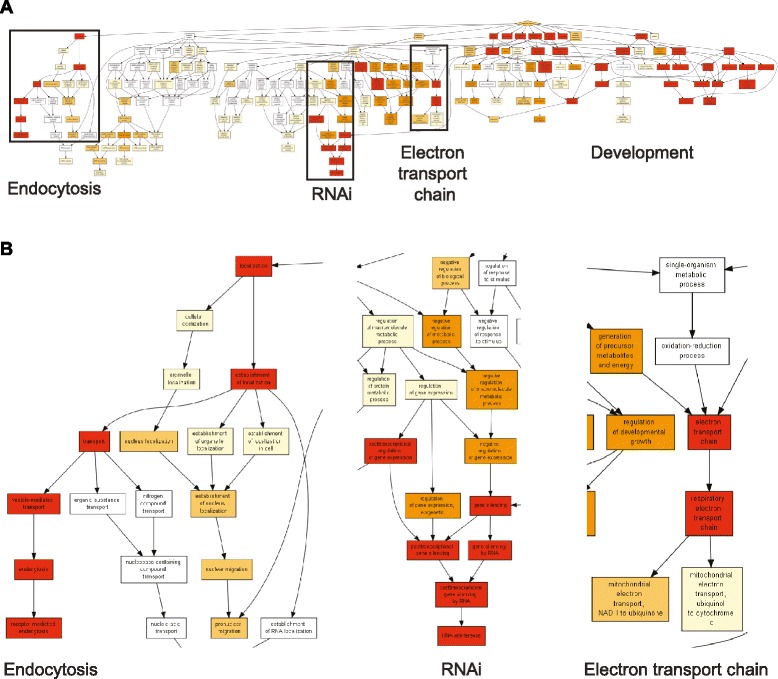
Enrichment of gene ontology terms for Nipi genes. Overview (**a**) and close-up views (**b**) of GOrilla analysis of Nipi genes. The color indicates the degree of enrichment, from red (very significantly enriched) to white (not enriched). The regions enlarged in b are indicated by the boxes in a. In addition to the three categories shown in b, in a, the other very significantly enriched processes, between “RNAi” and “Electron transport chain” are generic (“regulation of multicellular organismal process” and “positive regulation of multicellular organismal process”); those on the right are related to development. See also Additional file 7: Table S6

Most of the genes (245/278) were found in at least one significantly enriched class (Additional file 7: Table S6). The different classes were more or less related (Fig. 5); for example, genes associated with the stability, localization, and function of P granules (class 15 in Table 3) clustered with those associated with Rab11-positive recycling endosome-linked transport (class 10). Many genes belonged to several functional classes; the most frequently found (in 12/34 classes, Additional file 7: Table S6) encodes the E2 ubiquitin-conjugating enzyme LET-70. The most significantly enriched class was for genes defined as being conserved in *Drosophila* (through a pairwise comparison with *C. elegans*), followed by those previously described as stimulating microbial aversion behavior when knocked down by RNAi (classes 1 and 2 in Table 3, respectively). The latter includes genes involved in diverse essential cellular functions [26]. Several other classes linked to stress responses were also highly enriched. One of the most populated classes (50/278) was of genes previously characterized as being necessary for RNAi (class 8 in Table 3). This surprising result was corroborated by an analysis of enriched GO terms using GOrilla [48] (Fig. 6; Additional file 7: Table S6), and is discussed below. The proportion of non-histone MAPK signaling genes present in each class varied widely; for “microbial aversion” (class 2) it was 11/33, but was 0/33 for three classes (classes 5, 17 and 29; Table 3). This list of 33 MAPK-related genes overlapped well (8/33) with the list of genes in class 7, reported to be required for multiple cytoprotective responses (i.e., regulators of *gst-4* (detoxification), *hsp-4* (endoplasmic reticulum unfolded protein response (UPR)), *hsp-6* (mitochondrial UPR,UPR^mt^) and *sod-3* (reactive oxygen species (ROS) response) [49]). There was an equivalent overlap (8/33) with the targets of clones able to suppress over-expression of *gpdh-1p*::*gfp* seen in *osm-8* mutant worms (class 3). As discussed further below, this gives a further indication of the degree of imbrication of MAPK signaling with different cellular homeostatic processes.

**Fig. 7:**
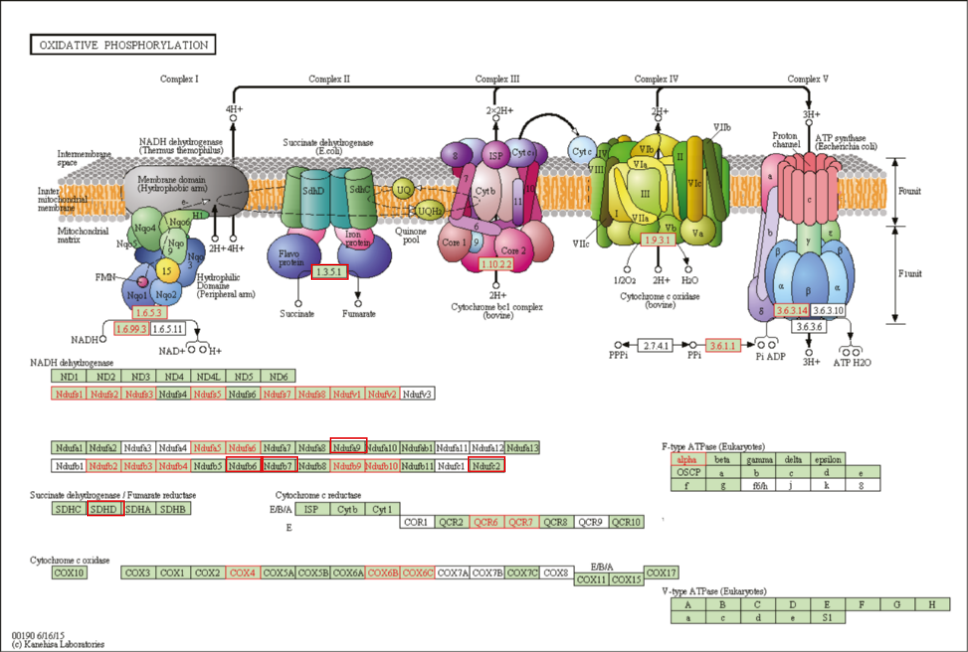
Participation of Nipi gene products in oxidative phosphorylation. The upper part of the figure shows a KEGG-derived schematic representation of the successive complexes that make up the mitochondrial electron transport chain (KEGG pathway cel00190) on which the complexes that include any proteins corresponding to Nipi clone targets are highlighted. For any targets of the 297 clones that did not provoke a strong developmental phenotype, the E.C. name for each complex is boxed in red with red text; for the targets of the remaining 63 clones (Table 2; Additional file 5: Table S5) there is one additional complex (E.C. 1.3.5.1, succinate dehydrogenase), boxed in red with black text. The lower part of the figure shows the individual protein components of the different complexes, annotated as above. Proteins for which there is no KEGG-assigned ortholog in *C. elegans* are uncolored. A full explanation of the symbols can be found at http://www.kegg.jp/kegg/document/help_pathway.html

We previously reported a potential role for endocytosis in the induction of *nlp-29* expression provoked by fungal infection [7]. Consistent with this, the GOrilla analysis (Fig. 6a; Additional file 7: Table S6), in common with EASE, also highlighted the role of endocytosis in the regulation of *nlp-29p::gfp* reporter gene expression. They also both drew attention to the potential role of mitochondria in regulating the innate immune response (Fig. 6 and Table 3). For example, all the genes present in at least three of the top four EASE functional classes encode mitochondrial proteins (Additional file 7: Table S6). A Kyoto Encyclopedia of Genes and Genomes (KEGG) analysis [50] of the targets of the 297 Nipi clones (Additional file 5: Table S5) assigned 22 to the category oxidative phosphorylation, corresponding to proteins present in four of the five complexes of the mitochondrial electron transport chain. Extending the analysis to include the targets of the 63 clones that provoked a severe developmental phenotype increased the total number of mitochondrial proteins to 27, covering all five electron transport chain complexes (Fig. 7). The role of mitochondria in the regulation of *nlp-29* expression is explored further below.

### Foundling and orphan genes

A total of 33 genes were not found to be associated with any EASE class (Table 5; Additional file 7: Table S6). A WormNet analysis failed to reveal any significant connection between the members of this group (area under the curve = 0.5; *P* = 0.3). Inspection of this list, however, revealed it to include two genes known to play specific and important roles in the regulation of antimicrobial peptide gene expression, namely *dcar-1* and *sta-2*, which encode, respectively, a DAMP receptor [20] and a STAT-like transcription factor [7]. Our EASE database [46], which currently contains more than 500 classes, has been built up by manual annotation and is necessarily biased to categories that we expect to be of interest in our studies. In an attempt to overcome this limitation, we assembled a far more complete and unbiased collection of functional classes, extracting data automatically from multiple sources including WormBase, FlyBase [51], KEGG [52], and the relevant RNAi databases [53, 54], and combined this with our EASE database to give a collection of more than 3700 classes of genes. Even using this collection, we failed to find any significant enrichment for the group of 33 genes.

**Table 5:** Foundling Nipi genes

Gene/sequence name	Brief description
*dcar-1*	GPCR receptor; activated by HPLA [20]
*elo-2*	Palmitic acid elongase; ELO-2 is required with ELO-1 for 20-carbon PUFA production
*frpr-11*	GPCR of the FMRFamide Peptide Receptor family; unlike *dcar-1*, acts downstream of *gpa-12* and blocks osmotic induction of *nlp-29* [20]
*ins-6*	Predicted type-beta insulin-like peptide
*mltn-12*	MLt-TeN (*mlt-10*) related; MLT-10 is a nematode-specific protein required for ecdysis
*nas-37*	Astacin-class metalloprotease required for ecdysis; N-terminal signal sequence followed by an Astacin protease domain and three protein-binding domains (EGF-like, CUB, and thrombospondin)
*srsx-25*	GPCR of the serpentine receptor class SX
*srv-21*	GPCR of the serpentine receptor class V; unlike *dcar-1*, acts downstream of *gpa-12* and blocks osmotic induction of *nlp-29* [20]
*sta-2*	STAT family of transcription factor [7]
F56A8.5	Protein containing an F-box
K08C9.5	Protein containing an F-box
C33D9.3	Nematode-specific
F27C8.2^a^	Nematode-specific
F27C8.3^a^	Nematode-specific
F34H10.1	Ubiquitin/40S ribosomal protein S27a fusion protein
K08C9.7	Ubiquitin/40S ribosomal protein S27a fusion protein
R186.8	39S ribosomal protein L33, mitochondrial
T08D2.2^b^	Similar to C-terminal half of UDP-N-acetylglucosamine-dolichyl-phosphate N-acetylglucosaminephosphotransferase
T08D2.6^b^	YIPF4-like; YIPF4, poorly characterized membrane spanning protein; in yeast, interacts with Rab GTPases
Y60A3A.19	YIPF4-like.;YIPF4, poorly characterized membrane spanning protein; in yeast, interacts with Rab GTPases
C42C1.3	Nematode-specific
F35F10.14	Nematode-specific
F41H8.1^c^	Nematode-specific domain: GPCR of the serpentine receptor class BC
K09C6.6^c^	Nematode-specific domain: GPCR of the serpentine receptor class BC
K09C6.10^c^	Nematode-specific
F42C5.9	Actin
F45E4.5	Nematode-specific
F49H12.5	Thioredoxin domain-containing protein 12-like
F52C6.13	Nematode-specific
*tsen-54*	N-terminal half similar to that of tRNA-splicing endonuclease subunit Sen54
Y39G10AR.7	Nematode-specific
Y51H7BR.7	Contains Spec3 domain, like *Drosophila* Stumbled
Y67D8C.22	Clarin-like

^a,b^Targeted by a single mv clone

^c^Potential targets of the same sjj clone

References are given for genes previous connected to the regulation of *nlp-29* expression; see Additional file 7: Table S6

Genes for which homologues are found only in a specific taxonomic group, irrespective of the level (e.g., animals, nematodes, or *Caenorhabditis*), are called taxonomically-restricted genes (TRGs). TRGs that are restricted to a very narrow taxonomic group, generally a species, can be called orphan genes [55]. Here, we have described a group of genes for which there is no pertinent functional data in a wide range of publically available databases. In a sense, it is as if these genes have been abandoned. By analogy with the term orphan gene, we apply here the term “foundling gene” to them.

**Fig. 8:**
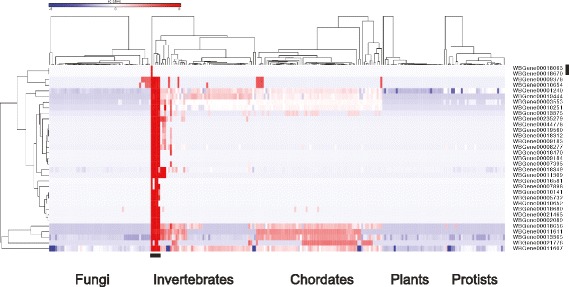
Phylogenetic profiles for 33 foundling genes. Hierarchical clustering of the normalized bit scores for homologs for 33 foundling Nipi genes across the genomes of 238 species present in Ensembl (see Additional file 8: Table S7 for the list of species in the order they appear here). For each of the groups (fungi, invertebrates, etc.), the species are clustered independently. The colour code reflects the relative normalized bit score, from high (red) to low (blue) across the different species. The horizontal bar at the bottom marks the position of the five *Caenorhabditis* species, from the left to right, *C. elegans*, *C. brenneri*, *C. briggsae*, *C. remanei*, and *C. japonica*. Several distinct groups of genes can be discerned, including genes unique to *C. elegans* (i.e., orphan genes [123]), indicated by the vertical bar on the right

The failure to connect the foundling genes could be because they are not in reality linked to each other in any way, or, the explanation we favor, they collectively play specific roles in nematode epidermal defense against fungal infection, which has not hitherto been sufficiently completely described. Such an idea is in line with the pattern of conservation of the 33 foundling genes; more than half are TRGs and encode proteins that are essentially restricted to nematodes. Others are present in a broad range of invertebrate and vertebrate species, while two (WBGene00018063 and WBGene00018670) are currently orphan genes, with no homologs outside *C. elegans* (Fig. 8; Table S7 ). Genes with similar phylogenetic profiles are more likely to function together in a common biological process [56, 57]. Thus, these diverse patterns of conservation will contribute to elucidating the function of these foundling genes.

### Epistasis and functional analyses with candidate clones

The enriched gene classes included four related to osmotic stress, corresponding to a total of 71 genes (Table 3; Additional file 7: Table S6), consistent with the previously established connection between osmotic stress and antimicrobial peptide gene expression [2, 8, 58]. To investigate this link further, we complemented our *in silico* analyses with direct assays to test the capacity of the 297 RNAi clones (Table 2) to block the increase in *nlp-29p::gfp* expression provoked by osmotic stress. Another enriched class was of genes that, when knocked down, provoke the expression of an *irg-1p::gfp* reporter in the nematode intestine [59]. This category is linked to innate immunity since *irg-1* encodes a putative antibacterial effector protein, induced in the intestine upon infection with pathogenic *Pseudomonas aeruginosa* by *zip-2*, which promotes defense [59]. The 297 clones were therefore assayed for their capacity to induce the expression of *irg-1p::gfp*. They were also used in a test of epistasis, by quantitating their potential for abrogation of the elevated *nlp-29p::gfp* expression associated with a constitutively active form of GPA-12 (GPA-12*), the alpha subunit of a heterotrimeric G protein that acts between DCAR-1 and TIR-1 [5].

More than one third of the clones tested (100/297, 34 %; termed here, “I-clones”) provoked a marked increase in *irg-1p::gfp*. When compared to the results of Dunbar *et al*. [59], there was a very satisfactory overlap, with identification of 31 of a possible 44 genes previously associated with the phenotype. This figure is similar to that reported by others when comparing screens performed in different laboratories (75 %; [60]). Importantly, many additional candidate negative regulators of *irg-1p::gfp* expression were identified (Additional file 5: Table S5). This is one of the positive consequences of our having conducted a quantitative screen. These results illustrate how the targets of the Nipi clones can have pleiotropic roles, being positive regulators of an epidermal AMP gene, but negative regulators of an intestinal defense gene. They also suggest that the reciprocal relationship between gene regulation in these two tissues that we found for *vhp-1* may reflect a more general phenomenon.

A high proportion (153/297; 51.5 %) of clones abrogated the high constitutive expression of *nlp-29p::gfp* seen in a strain expressing GPA-12* (“G-clones”). Since the p38 MAPK cassette functions downstream of *gpa-12* in the regulation of *nlp-29p::gfp* [7, 9], clones targeting components of the MAPK signaling cascade would be expected to be especially well-represented in this category. In fact, this was not the case, as only 18 of the 43 clones (42 %) were G-clones (Additional file 5: Table S5). This could be interpreted to mean that a substantial number of G-clones provoked the phenotype for relatively non-specific reasons. Indeed, of the 153 G-clones, 104 (68 %) were found to block the induction of *nlp-29p::gfp* normally provoked by osmotic stress, a markedly higher number than expected, since, in the complete set of 297 clones, there were 131 (44 %) in this category (“O-clones”; Additional file 5: Table S5). Further, 21 of the G-clones targeted genes required for the expression of an *acdh-1p::gfp* reporter gene (class 4, Table 3); *acdh-1* encodes a key enzyme in fatty acid metabolism. Finally, a quarter of the targets of the O-clones (37/147) had previously been associated with the response of *C. elegans* to osmotic stress (Additional file 7: Table S6). The significance of these different overlaps is discussed below.

As described above, an alteration of fungal spore adhesion can lead to a change in defense gene expression. Assaying spore adhesion to worms cultured on RNAi clones proved experimentally challenging because of the variable phenotypes routinely seen with RNAi and especially since there were so many clones to test. We did, however, identify 12 clones that appeared to affect, to a greater or lesser degree, this initial step of the infection process. We were surprised to discover that six were G-clones and five were O-clones, suggesting that the target genes might well play an additional role in governing *nlp-29p::gfp* expression (Additional file 9: Table S8). As a consequence, we did not remove these clones (representing < 5 % of the total) from our lists.

### Conserved protein complexes

**Fig. 9:**
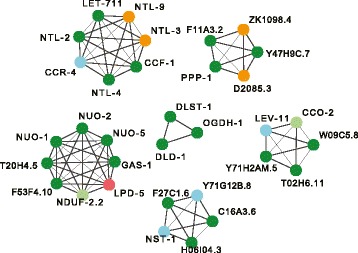
Enrichment of proteins encoded by Nipi genes in selected protein complexes. The components of several known complexes are shown, namely, clockwise from top left, with the complex ID(s) in brackets (Additional file 10: Table S9), CCR4-NOT (YChrMod1004), eukaryotic translation initiation factor 2B complex (195; AMMC1231), mitochondrial ETC (218; AMMC1876), 66S pre-ribosomal particles (169; AMMC1267), mitochondrial complex I (65; AMMC1203), and in the center, mitochondrial oxoglutarate dehydrogenase complex (OGDC; YPC_CON1369). Proteins in dark green correspond to members of the final set of 338 Nipi genes, those in light green and orange to the presumed targets of clones that passed the first round of selection or that gave a single positive result in the first round, respectively. The clone that potentially knocks down *lpd-5* (corresponding protein in red) abrogated *nlp-29p::gfp* expression but had a marked effect on development and reduced *col-12p::dsRed* expression. The remaining proteins are shown in blue

Functional modules frequently correspond to physical protein complexes. Several studies have defined a variety of protein complexes from different species. One recent report provided more than one million putative high-confidence co-complex interactions present broadly across animal species [61]. Combining this with data from yeast [62–64], and having identified the *C. elegans* orthologues of the component proteins when necessary, we compiled a collection of 1925 predicted *C. elegans* protein complexes (Additional file 10: Table S9). We then associated each of the predicted targets of the Nipi RNAi clones with the different complexes. We focused on complexes with at least three components for which we had picked up more than half of the components in our screen (Additional file 10: Table S9). There was an over-representation of the eukaryotic translation initiation factor (eIF) 2B complex and 66S pre-ribosomal particles, suggesting an important role for protein translation. There was also enrichment for components of the carbon catabolite repression 4-negative regulator of transcription (CCR4-NOT) complex, which is a major mRNA deadenylase, linked to mRNA degradation and general transcriptional regulation, among other functions [65]. We discuss these observations below. The analysis also indicated that there was enrichment in several mitochondrial complexes (Fig. 9). This is consistent with the KEGG analysis described above (Fig. 7), and we focused our attention on this class of gene.

### Intestinal UPR^mt^ inhibits epidermal AMP expression

A total of 30 genes identified in our screen have been shown to induce a mitochondrial UPR (UPR^mt^) when inactivated [66]. For example, the well-characterized *spg-7* that encodes a mitochondrial metalloprotease, was picked up with two independent RNAi clones in our screen (Additional file 5: Table S5). This suggested that activation of the UPR^mt^ could block the expression of antimicrobial peptide genes in the epidermis. At the same time, in contrast to intestinal infection with *P. aeruginosa* [67], infection of young adult *C. elegans* by *D. coniospora* does not provoke the UPR^mt^ since the expression of the hallmark genes *hsp-6* and *hsp-60* is unchanged [2, 46].

**Fig. 10:**
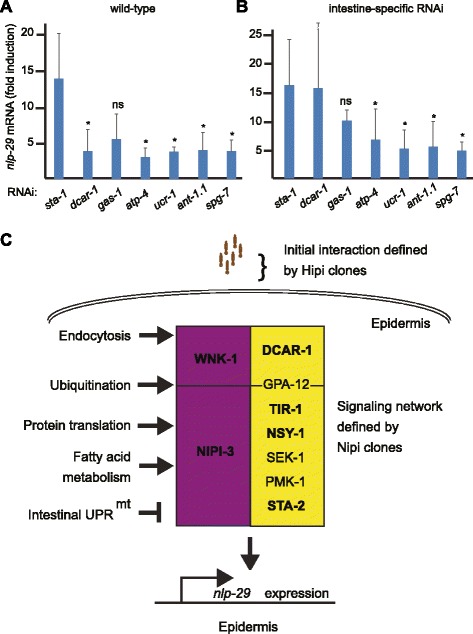
Cell autonomous and non-autonomous mechanisms influence *nlp-29* expression. Quantitative RT-PCR analysis of *nlp-29* gene expression level showing the infection-associated “fold induction” (infected/non-infected values) in worms treated with positive and negative RNAi control clones (targeting *dcar-1* and *sta-1*, respectively) or clones that provoke a UPR^mt^ (targeting *ant-1.1*, *atp-4*, *spg-7*, *ucr-1*, and *gas-1*) in a wild-type (**a**) or the intestine-specific RNAi strain MGH171 (**b**) for 30 h before infection with *D. coniospora* for 18 h. Results are the average (± SD) from four and three experiments, respectively (see Additional file 11: Table S10). The difference between control and *gas-1*(RNAi) is not significant (ns) in either strain; * *P* < 0.05 (unpaired *t*-test). The SD for *dcar-1* in (b) is 14. **c** Simplified model of pathways and processes involved in the regulation of *nlp-29*. The screen identified Hipi genes that modulate the adhesion of spores to the worm cuticle, and Nipi genes (central box) required for the expression of *nlp-29* upon osmotic stress and infection (purple), or only after infection (yellow), acting downstream (below horizontal line) or upstream of or parallel to GPA-12. Only a very limited number of genes are shown; those in bold were identified in the screen. The Nipi genes fall into multiple functional categories; some are listed on the left, positioned arbitrarily; pointed and flat arrows indicate positive and negative regulation, respectively

Given the links that exist between the UPR^mt^ and antibacterial defenses in *C. elegans* [68], we decided to explore in more depth the relationship between the response to fungal infection and the UPR^mt^. As a first step, to validate the results of the screen obtained with the reporter construct, we used qRT-PCR to assay the level of the endogenous *nlp-29* transcript following knock-down of five candidate genes, all associated with the activation of other stress reporter transgenes (Additional file 7: Table S6), including *spg-7*, a well-established means of inducing the UPR^mt^ [69]. Inactivation of four of them (*ant-1.1*, *atp-4*, *spg-7*, and *ucr-1*) abrogated *nlp-29* gene expression after infection to the same degree as the positive control, *dcar-1*, while knocking down *gas-1* did not have a statistically significant effect (Fig. 10a).

In *C. elegans*, the UPR^mt^ can involve trans-tissue signaling (reviewed in [12]). Thus, for example, provoking an UPR^mt^ just in neurons leads to an UPR^mt^ in the intestine [70]. To address the question of whether the inhibitory effect of the UPR^mt^ on *nlp-29* gene expression might also be cell non-autonomous, we assayed the effect of knocking down the same five candidate genes by RNAi specifically in the intestine in the strain MGH171 [26]. In this case, in contrast to intestinal knockdown of *dcar-1*(RNAi), which gave the same average level of expression of *nlp-29* as *sta-1*(RNAi), consistent with *dcar-1*’s cell autonomously function [20], intestinal RNAi of *ant-1.1*, *atp-4*, *spg-7*, and *ucr-1* was associated with an abrogation of *nlp-29* gene expression following *D. coniospora* infection, while *gas-1*(RNAi) again did not provoke a statistically significant effect (Fig. 10b). Overall, our results indicate that provoking the UPR^mt^ in the intestine reduces the induction of an antimicrobial peptide gene in the epidermis (Fig. 10c).

## Discussion

### Qualitative versus quantitative RNAi screens

Genome-wide RNAi screens have been performed in *C. elegans* for more than a decade. Their experimental basis is relatively straightforward, since RNAi by feeding is an effective technique in worms [71]. In a number of cases, the read-out has been the effect of RNAi on the expression of a reporter gene or the localization of a chimeric reporter protein, to address a specific biological question (e.g., [49, 57, 59, 66, 72–77]). Generally, these have been visual screens. Despite certain advantages [78], visual screens include an element of subjective judgment, lack discriminatory power, and are best suited to identifying clones that provoke a marked phenotype. These will generally target genes at the central nodes of a signaling network. Full understanding of regulatory mechanisms also requires, however, the identification of genes that exert only a minor effect [23].

An alternative is to undertake automated quantitative screens. These require specialized equipment and tools for data storage and analysis [18, 19, 28, 79–82] and are thus more difficult to put in place. Further, they also suffer from the intrinsic variability of RNAi, which cannot be adequately accounted for using formal statistical analyses (Thomas Richardson, University of Washington, personal communication). Coupled with the continuous distribution of the results, this renders the definition of candidates somewhat arbitrary. In this study, as discussed in more detail elsewhere [83], we used the results for clones targeting genes known to be important for the regulation of *nlp-29* to establish cut-offs, and in the first round of screening for Nipi clones, we privileged those giving a reproducible effect. Similarly, the cut-offs we used to identify Peni and Hipi clones were based more on criteria of reproducibility rather than strict statistical criteria. The thresholds we adopted will necessarily determine the candidate genes identified, and could bias our global analyses. We have provided, however, for the first time, via a dedicated web interface, the complete set of results for the two rounds of selective screening, measuring multiple parameters for individual worms from each population. Not every clone in the library will contain the expected insert. Extrapolating from our sequencing of 388 clones, which revealed an error rate of 14 %, around 3000 clones in the complete library might be incorrect. With this caveat in mind, the complete set of results contains a substantial amount of information that we have not attempted to exploit, for example, linked to inter-individual variability of gene expression, terminal epidermal cell fate determination, or simply genes that affect the development and size of *C. elegans*. It also will be an important resource for those wishing to develop new analytical methods; these would be required to leverage the intrinsically variable quantitative data for subsequent analyses.

### Genes affecting spore adhesion

One class of genes to emerge from this screen is the Hipi genes that affect the initial adhesion of *D. coniospora* spores to *C. elegans*. This requires contact between the spores’ adhesive bud and the outermost layer of the nematode cuticle, the surface coat. In contrast to the underlying collagen- and cuticlin-rich cuticle, the surface coat is rich in structural glycoproteins, including mucins [84, 85]. Two of the candidate Hipi genes, *bus-2* and *bus-12*, were known to affect the surface coat. Their role in adhesion of *D. coniospora* has been investigated using the corresponding mutants [31]. These were originally isolated because, unlike wild-type worms, they were not susceptible to infection by the bacterial pathogen *Microbacterium nematophilum* that normally adheres to specific areas of the worm cuticle [86]. Both genes are important for the post-translational modification of surface-exposed proteins [29, 30]. Several other Hipi genes encode conserved enzymes. Their precise role in mediating spore adhesion will require detailed study but, as mentioned above, they too might alter surface protein maturation. Another candidate, K06A9.1, is nematode-specific. It is predicted to encode several protein isoforms, including one of more than 2200 amino acids, comprising 22 degenerate 81 amino acid repeats. Taken together with its distant similarity to mucins, this suggests that it could be a component of the surface coat.

The gene *ykt-6* encodes the worm ortholog of Ykt6p, a v-SNARE essential for endoplasmic reticulum-Golgi transport [87]. It could be required for the correct transport of surface proteins. On the other hand, *ykt-6* has been linked to insulin signaling in *C. elegans* [88] and, interestingly, *ins-6*, which encodes an insulin-like peptide, was identified as a Nipi gene in the current screen (Table 5). An *ins-6* loss-of-function mutant, however, did not display a Nipi phenotype (unpublished results). A lack of concordance between phenotypes observed using RNAi and mutant strains has previously been reported (e.g., [89]). The definitive attribution for a role in spore adhesion for the various Hipi genes must therefore await individual genetic validation.

### MAPK signaling and osmotic stress responses

The results of the screen reaffirm the central place of p38 MAPK signaling in the regulation of the *nlp-29* AMP gene in the epidermis [90] and substantially expand the catalog of genes involved. Many of the same genes are also required not only for xenobiotic detoxification, the UPR, the UPR^mt^, and the response to ROS [49], but also for the regulation of *gpdh-1*, a gene that encodes the rate-limiting enzyme in the biosynthesis of the osmoprotectant glycerol [72, 91]. The expression of *gpdh-1* does not change following infection with *D. coniospora* [2, 46], but is elevated upon exposure to high concentrations of salt, via a mechanism that involves inhibition of translation. This is mediated by the general control non-derepressible (GCN-2) kinase signaling pathway that controls eIF-2α phosphorylation and the activity of the with-no-lysine kinase and Ste20 kinases WNK-1 and GCK-3 [92]. In contrast, the results of our screen amply demonstrated that inhibition of translation does not activate *nlp-29* expression. Quite the contrary, many clones targeting genes required for translation, including those encoding aminoacyl-tRNA synthetases (*hars-1*, *lars-1*, *rars-1*, *tars-1*, *wars-1*) and eIF subunits, were required for expression of *nlp-29* after infection. Although we have not yet determined whether these effects are cell autonomous, this AMP gene therefore distinguishes itself from effectors of other stress responses, such as *irg-1* and *gpdh-1*. On the other hand, its expression upon osmotic stress does require *wnk-1* and *gck-3* [8], which is also the case for *gpdh-1* [92]. We show here that the induction of *nlp-29* expression after infection also requires *wnk-1*, acting upstream or in parallel to *gpa-12* (Additional file 5: Table S5) and to a lesser extent *gck-3* (Fig. 1b). Further, in common with two thirds of the genes that act downstream of *gpa-12* (the targets of the G-clones, including *hars-1*, *lars-1*, *rars-1* and *tars-1*), we found that *nipi-3*, which encodes a homolog of Tribbles required for the response to infection [3], is also required for the expression of *nlp-29* upon osmotic stress. These results lead to a revision of the infection/osmotic stress dichotomy [8] of our previous models for the regulation of *nlp-29* (Fig. 10c).

### Cross-tissue communication

One unexpected finding regarding MAPK signaling was that *vhp-1*(RNAi) abrogates *nlp-29* expression, since VHP-1 has been described as a negative regulator of p38 PMK-1 in the nematode intestine [43, 93]. There, the p38 MAPK has a well-characterized role in defense against bacterial pathogens that colonize the gut lumen. We have previously observed that there is an overrepresentation among the genes induced by *D. coniospora* of genes repressed after infection by the bacteria *S. marcescens*, *E. faecalis*, and *P. luminescens*. This enrichment includes numerous antimicrobial peptide genes of the *nlp* and *cnc* classes [46]. In other words, bacterial infection of the gut, which switches on the p38 MAPK pathway, implicating a decrease in VHP-1 activity, abrogates epidermal antimicrobial peptide gene expression. Our current hypothesis is that *vhp-1*(RNAi) activates the p38 MAPK pathway in the intestine, and that this has the paradoxical consequence of reducing p38 MAPK activity in the epidermis despite a reduction of *vhp-1* expression in that tissue too. We currently have no plausible explanation for the observation of ectopic, intestinal expression of *nlp-29* following knockdown of *vhp-1* only in the epidermis, but note that other analogous examples of cell non-autonomous regulation have been recently reported [78].

A further example of communication between tissues was revealed in our investigation of the impact of the UPR^mt^ on AMP gene expression. Knocking-down, specifically in the intestine, one of several genes known to trigger an UPR^mt^ caused a reduction of *nlp* gene expression following *D. coniospora* infection. Recent studies have suggested that intestinal pathogens can provoke the UPR^mt^ in *C. elegans* and that this switches on defense gene expression in the intestine [67, 74]. The UPR^mt^ is negatively regulated by the Jun kinase KGB-1 [94], which in turn is negatively regulated by VHP-1 [43, 93]. While this mechanism is complemented by another pathway involving ROS-stimulated eIF2α kinase that leads to a reduction in protein translation [95], compromising overall translatory capacity can by itself cause the expression of defense genes such as *irg-1* [59, 96] and *gpdh-1* [92], but prevents ROS-induced UPR^mt^ [94]. While the precise interplay between this complex series of homeostatic and cellular defense mechanisms is far from being understood [12, 97], these different findings are compatible with a model wherein activation of anti-bacterial defense mechanisms in the intestine, whether directly upon infection with bacterial pathogens, by reducing VHP-1 activity, by reducing protein translation, or following a UPR^mt^, leads to a suppression of the capacity of the epidermis to express antifungal defense genes. As such, this could constitute a mechanism to ensure an appropriate allocation of resources within the organism, with the aim of concentrating energy to defend one tissue, to the detriment of the capacity of the epidermis to express AMPs.

### Fatty acid metabolism and AMP gene expression

Previous studies have suggested a possible link between fatty acid metabolism and innate immunity in *C. elegans* [8, 27, 98]. This is further reinforced by the fact that both *dld-1* and *elo-2*, respectively encoding a dihydrolipoamide dehydrogenase and a palmitic acid elongase, were identified as Nipi genes in our screen. Further, the expression of *acdh-1* and *acdh-2*, which encode mitochondrial short-chain acyl-CoA dehydrogenases that catalyze the first step of fatty acid beta-oxidation, is markedly reduced when *C. elegans* is infected either with *D. coniospora* or with a number of different bacterial intestinal pathogens [46, 99]. The *elo-2* paralog *elo-3* was previously found to be required for the expression of *acdh-1p::gfp* [73]. A total of 35 Nipi genes are also regulators of *acdh-1p::gfp* expression (Table 3). These include the mediator complex gene *mdt-15*, a major regulator of fatty acid metabolism and longevity [100, 101]. MDT-15 is also required for oxidative stress responses and the induction of specific detoxification genes in response to xenobiotics or heavy metals [102–104]. MDT-15 was recently shown to have a more direct role in innate immunity since it regulates the expression of p38 MAP kinase PMK-1-dependent immune genes and resistance to *P. aeruginosa* infection [105]. The links between fatty acid metabolism and host defense in *C. elegans* clearly merit more detailed investigation.

### Further functional groups involved in AMP gene expression

Several other groups of functionally related genes were also identified among the Nipi genes. Almost 50 genes had previously been characterized as being necessary for transgene silencing [57]. This is paradoxical since, if RNAi were not efficient in our system, we would not expect an RNAi-dependent reduction in reporter gene expression. Many of the genes in this category clearly play an indirect role in transgene silencing. To give just one example, *dpy-4* encodes a cuticle collagen and is required for normal morphology. Whether they play direct roles in modulating *nlp-29p::gfp* expression remains to be established.

There was a similar overlap of Nipi genes with genes required for the correct sub-cellular localization of the RAB-11, a small GTPase involved in endocytosis, and for transport of the apical membrane protein PEPT-1 [79]. This is consistent with our observation that knocking down dynamin (encoded by*dyn-1*), which is involved in the scission of newly formed clathrin-coated endocytic vesicle from the cell membrane, or the small GTPase Rab5 (*rab-5*), which characterizes early endosomes derived from dynamin-dependent and independent endocytosis, abrogates *nlp-29* gene expression after infection [7]. Endosomal membranes may function as important platforms for innate immune signaling in *C. elegans* as in other species [106, 107].

Among the three Nipi genes (*icd-1*, *let-70*, and *let-92*) required both for transgene silencing [57] and endocytosis [79], as mentioned above, the E2 ubiquitin conjugating enzyme gene *let-70* is linked to a broad range of cellular and organismal functions. It is noteworthy that we found nine other genes involved in ubiquitination and proteasome-mediated protein catabolism (*dcaf-1*, *hecd-1*, *pas-3*, *pas-5*, *pbs-2*, *prp-19*, *rbpl-1*, *skr-1*, *usp-39*) in the present screen. It is likely therefore that, in *C. elegans*, ubiquitination plays an important role in regulating innate immune responses, as it does in many species by governing the stability of key signaling molecules [108–111].

Finally, there was enrichment for components of the CCR4-NOT complex. This complex coordinates a variety of cellular processes, acting at all levels of gene expression, including transcription and mRNA or protein stability. It is involved in cellular adaptation to external stress, including the control of the vertebrate innate immune response through the regulation of STAT1 [112]. It may act in a similar manner to influence the activity of the STAT-like transcription factor STA-2 and thereby the expression of AMP genes in *C. elegans*.

### Conservation and innovation in innate immune defenses

In nature, infection represents an extremely strong selection pressure. This is reflected by the evolution of sophisticated host defense mechanisms, driven by the different pathogens that exercise a negative impact on fitness and survival in the environment. In jawed vertebrates, this has led to the emergence of the adaptive immune system, based on a specific collection of genes and mechanisms not found outside the infraphylum [113–115]. Similar specialization involving groups of TRGs involved in immunity is observed in other branches of the animal kingdom [116]. Here, we identified a number of genes required for the expression of an antimicrobial peptide in *C. elegans* that are restricted to nematodes. They are expected to be part of a lineage-specific defensive innovation. Their further study will contribute to our understanding of the evolution of immunity in *C. elegans* [117, 118]. Our results also highlighted the links that exist between antimicrobial defenses and the homeostatic mechanisms that counter abiotic stress. This supports an ancient origin for the co-adaptive evolution of stress and innate immune responses (e.g., [119]).

## Conclusions

In conclusion, this genome-wide study has allowed the identification of hundreds of genes that modulate the capacity of *C. elegans* to express the AMP gene *nlp-29* following infection with *D. coniospora*. Not only has it greatly expanded the number of such Nipi genes, but it has also revealed multiple interwoven cellular regulatory mechanisms that impinge on AMP gene expression. Understanding the precise nature of the regulatory activity exercised by the Nipi genes in each of these different functional classes, as well as the many individual genes, will require focused study in the future.

## Methods

### Nematode strains

All strains were maintained on nematode growth media and fed with *E. coli* strain OP50. The strain for intestine-specific RNAi, MGH171 *sid-1*(*qt9*); *alxIs7*[*vha-6*p::SID-1::SL2::*gfp*] [26] was kindly provided by Justine Melo and the strain AU133 *wt*; *agIs17*[*irg-1*p::*gfp*;*myo-2*p::*mCherry*] [96] by Emily Troemel. Details about the constructions of the strains IG274 (*frIs*7[*nlp-29*p*::gfp*, *col-12*p*::DsRed*] *IV*), IG1389 (*frIs7*; *frIs30*[*col-19*p::gpa-12*,*unc-53*pB*::gfp*] *I*), and the epidermis-specific RNAi strain IG1502 (*rde-1(ne219) V; ls[wrt-2*p*::RDE-1::unc-54 3’utr; myo-2*p*::RFP3] III; frIs7 IV*) are provided elsewhere [3, 5, 20].

### A genome-wide RNAi library

In order to cover the maximum number of target genes, as specifically as possible, we combined RNAi clones from the Ahringer genomic [21] and Vidal cDNA [22] libraries. The constitution of the RNAi library was based on the data and tools for target prediction available at the time (WormMart WS220; now retired). If an Ahringer library clone was predicted by WormMart to have more than one primary target, when possible, we added Vidal library clones predicted to target individually any or all of the multiple primary targets. The Ahringer clones were directly redistributed from each 384-well library plate among four daughter 96-well plates. Of the 16,744 clones in our copy of the Ahringer library, 625 failed to grow. We equally sought to replace them with clones from the Vidal library and cherry-picked a total of 5136 clones. Of these, 32 failed to grow, leaving us with a collection of 21,223 clones (Additional file 1: Table S1). Among them, 132 Ahringer clones were present in two wells, so that the combined library included clones in 21,355 wells. This library of 21,355 wells was used in the first round of screening.

### Target prediction

Because of limitations in the method used by WormBase to predict the targets of an RNAi clone, as part of this project, we developed the tool CloneMapper [28]. Out of the 21,223 clones, 20,025 were present in CloneMapper, and were predicted to target 16,565 genes (score ≥ 1). For the remaining 1198 clones, despite the known shortcomings [28], we used WormMart WS220 and WormBaseConverter [46] (WS220 to WS240) to identify a further 1304 targets. Combined, the clones are predicted to target 17,415 of the 20,540 protein coding genes (84.8 %) in WS240.

### High-throughput RNAi screen

The RNAi screen was performed as previously described in detail [19, 83]. Briefly, synchronized L1 larvae were deposited in 96-well plates containing nematode growth media agar, with a different RNAi clone in each well. After 30 hours at 25 °C, when worms had reached the L3-L4 stage, a fresh solution of *D. coniospora* spores was added to each well, and worms were harvested 18 hours later for analysis using the COPAS Biosort. All data was stored in a custom-made database (Modul-Bio, Marseille, France) for subsequent analysis. Evaluation of the capacity of RNAi clones to block the increase in *nlp-29p::gfp* expression provoked by osmotic stress was performed as described [9]. Briefly, following culture on RNAi clones for 48 h, young adult worms were transferred into 96-well U-bottom plates containing 200 μL of 300 mM NaCl and gently agitated for 3 hours at 25 °C before Biosort analysis. Generally, a minimum of 80 synchronized worms were analyzed for size (TOF), extension, and green (GFP) and/or red (dsRed) fluorescence [18]. The inserts of candidate clones were sequenced to establish their identity.

### Data analysis and clone selection

Data analysis was performed as previously described in detail [19, 83]. Briefly, in the first round (whole genome) screen, for each well, a mean value for the GFP/TOF ratios for each worm was calculated. From these values, for each plate, a truncated mean (discarding the 25 % lowest and the 25 % highest values) was calculated and used to normalize the average GFP/TOF values for the individual wells, to allow across-plate comparison. Normalized values for TOF (TOF/[truncated mean of TOF]) and dsRed ((dsRed/TOF)/[truncated mean of dsRed/TOF]) were similarly calculated. Details of Nipi clone selection after the second round of screening are given in Supplementary Methods.

### Validation of the RNAi screening approach

A full description of the experimental validation of the screening approach can be found in a publicly available PhD thesis [83]. Of note, using the standard feeding protocol, a substantial number of RNAi clones can provoke severe developmental delays and/or larval lethality [21]. In an attempt to circumvent this, we transferred worms from their standard *E. coli* OP50 diet to RNAi bacteria at the early L3 stage and assayed the same worms when they were adults. Unfortunately, this was not a sufficiently robust method since, of the sequence-verified positive controls we tested, namely *pkc-3*, *rack-1*, and *sta-2*, only *sta-2* gave a phenotype [83]. Trying to increase the efficiency of the RNAi by using the RNAi sensitive strain *rrf-3* was also unsuccessful [83] since expression from high-copy transgenes is compromised in this background [120].

### Analysis of spore adhesion

Worms treated with each of the 28 Hipi clones from the L1 stage were infected as L4s with *D. coniospora*. To directly correlate spore adhesion and reporter gene expression, worms in the population (*n* ≥ 30) were visually inspected for their GFP expression, before accessing the adhesion of spores. A score was assigned, taking into account the intrinsic variability in GFP expression associated with infection (see, for example, Fig. 10b). Clones associated with a very high and homogenous induction were assigned a score of 2, clones associated with an induction similar to wild type were assigned a score of 0, and clones associated with an intermediate phenotype assigned a score of 1. Worms were then harvested in 50 mM NaCl, 0.05 % Triton, transferred to 96-well round-bottom well plates, and frozen at –80 °C. Plates were subsequently thawed and the number of spores attached to worms were counted at 230× using a Leica MZ16 stereomicroscope. Clones were assigned to three broad categories, relative to *sta-1*(RNAi)-treated control worms: 0 = 1–10 spores/worm (same as control); 1 = 10–25 spores; 2 = > 25 spores. A minimum of 30 animals were scored for each clone. Worms treated with the candidate Nipi clones from the L1 stage and infected as L4s with *D. coniospora*, as above, were analyzed slightly differently. The major part of each sample was analyzed with the Biosort, as above, and for the remainder, the number of spores attached to worms, at the head and vulva, were counted. An adhesion index was calculated: ((number of worms with *n* > 1 spores at the mouth) + (number of worms with *n* > 1 spores at the vulva))/(2 × total number worms). Clones associated with a score inferior to that of all control clones, and with a reduction of reporter gene expression greater than 50 % (i.e., loss of spore adhesion was accompanied by a reduction in the observed innate immune response) were selected. Clones selected in both duplicate tests were retained.

### Bioinformatic analyses

All analyses used the WS240 WormBase release, unless otherwise stated. Programs were written in Perl and the user interface was developed using HTML, PHP, JavaScript, and MySQL. We used WormNet v3 [42], EASE 2.0 [45] with an in-house database of functional annotations [46], GOrilla [48] with the November 2015 data update, and KEGG [52] release 77.1. For clustering, we used “One minus Pearson correlation” distance matrices within GENE-E (www.broadinstitute.org/cancer/software/GENE-E/).

### Data collection

In addition to the previously collected datasets for *C. elegans* functional classes used in EASE analyses and manually assembled from the literature, including differential transcriptomic and proteomic data, miRNA targets, TF targets etc., further classes were defined using data from a variety of resources.

1. We extracted all 1094 phenotypes available in WormBase WS246 from ftp://ftp.wormbase.org/pub/wormbase/.
2. A total of 1221 expression cluster datasets (WS246) were downloaded from ftp://caltech.wormbase.org/pub/wormbase/spell_download/.
3. We extracted the full list of *Drosophila* phenotypes from FlyBase release FB2014_06 via http://flybase.org/.bin/cvreport.html?cvterm=FBcv:0000347+childdepth=2, and manually collated closely related classes to give a list of 145 to which we matched the corresponding FlyBase Gene IDs. Using the DRSC Integrative Ortholog Prediction Tool (DIOPT) (http://www.flyrnai.org/cgi-bin/DRSC_orthologs.pl), we identified the *C. elegan*s orthologs for these genes. If more than one ortholog for a given fly gene was predicted, using an in-house perl script, we selected a best hit if the difference in the DIOPT score was greater than or equal to 2 (maximum score 10), but otherwise did not retain a worm ortholog.
4. Fly RNAi screen data was taken from http://www.flyrnai.org/RNAi_all_hits.txt (downloaded 14-11-14). RNAi clones for which no target gene was listed were excluded. Prediction of *C. elegans* orthologs was as above.
5. We downloaded all RNAi screen datasets for *Drosophila* from version 13 of GenomeRNAi.org [54] and manually collated very similar classes to give 110 datasets. Prediction of *C. elegans* orthologs was as above.
6. We extracted the genes corresponding to 136 KEGG_pathways (July 2015) and then converted identifiers to WormBase GeneIDs.

The resulting datasets are available on request.

A separate collection of predicted *C. elegans* protein-protein complexes was also assembled using experimentally determined protein complexes from other species [61–64]. Prediction of *C. elegans* orthologs was as above except that, if the difference in the DIOPT score was less than or equal to 2, the top two putative orthologs were retained.

### Phylogenetic profiling

To construct phylogenetic profiles, we followed an approach somewhat similar to that of Tabach et al. [57]. We collected data for a wide range of eukaryotic species. We downloaded the complete set of predicted proteins for 66 vertebrates from Ensembl (release 78) and for 55 invertebrates, 53 fungi, 32 plants, and 32 protists from Ensembl genomes (release 25). As many genes have multiple isoforms (e.g., 30,939 for 20,493 protein-coding genes in *C. elegans*), we chose the longest transcript for each gene. We used BLASTP to compare the proteins predicted for the 33 *C. elegans* foundling genes against all 238 proteomes and we chose the best hit for each. From this, we generated a “BestHit” matrix (33 × 238), where each entry C_ij_ is the best BLAST bit score of the top hit in species “j” for *C. elegans* protein “i”. As BLAST bit score depends on protein length, we normalized each bit score by calculating a self-similarity score C_ii_ (by BLASTing each *C. elegans* protein C_i_ against itself). We generated a normalized matrix by replacing each C_ij_ by C_ij_/C_ii_.

### RNA preparation and quantitative RT-PCR

RNA preparation and quantitative RT-PCR were as described [3]. Results were normalized to those of *act-1* and were analyzed by the cycling threshold method. Control and experimental conditions were tested in the same ‘run’. Each sample was normalized to its own *act-1* control to take into account age-specific changes in gene expression. Primers used for qRT-PCR are for:

- *act-1*: JEP538 ccatcatgaagtgcgacattg JEP539 catggttgatggggcaagag;
- *nlp-29*: JEP952 tatggaagaggatatggaggatatg JEP848 tccatgtatttactttccccatcc.

## Availability of data and material

The entire dataset for the first and second rounds of the RNAi screen are publically available at http://bioinformatics.lif.univ-mrs.fr/RNAiScreen/. Data from qRT-PCR experiments are provided in Additional file 11: Table S10. Custom programming scripts are available on request.

## Additional files

Additional file 1: Table S1. The list of 21,223 clones used in this study. (XLSX 355 kb) Additional file 2: Table S2. Results for Hipi clone candidates retained in the first round of screening and identification of potential target genes. (XLSX 134 kb) Additional file 3: Table S3. Results for Hipi clone candidates in the second and third round of screening; categorization of Hipi and Peni clones; clone verification and identification of potential target genes. (XLSX 112 kb) Additional file 4: Supplementary Methods. Details of how Nipi clones where selected after the second round of screening. (PDF 60 kb) Additional file 5: Table S5. Clone verification and the identification of potential target genes for 360 Nipi clones; genes that are potentially targeted by more than one clone; identification of clones potentially targeting histone genes; phenotypic characterization; comparison with published data. (XLSX 211 kb) Additional file 6: Table S4. Results for final 360 Nipi clone candidates in the first and second round of screening; identification of clones provoking severe developmental effect. (XLSX 1068 kb) Additional file 7: Table S6. WormNet and expression analysis systematic explorer (EASE) analyses. (XLSX 131 kb) Additional file 8: Table S7. Species used in phylogenetic clustering listed in the same order as in Fig. 8. (XLSX 47 kb) Additional file 9: Table S8. Analysis of spore adhesion following RNAi treatment with Nipi clones; identification of genes potentially involved in spore binding. (XLSX 132 kb) Additional file 10: Table S9. Analysis of enrichment of predicted Nipi proteins in conserved protein complexes. (XLSX 136 kb) Additional file 11: Table S10. Data from qRT-PCR experiments. (XLSX 22 kb)

## Abbreviations

AMP: Antimicrobial peptide
CCR4-NOT: Carbon catabolite repression 4-negative regulator of transcription
cnc: Caenacin (*Caenorhabditis* bacteriocin)
DIOPT: DRSC integrative ortholog prediction tool
DRSC: Drosophila RNAi screening center
DsRed: Red fluorescent protein from *Discosoma* sp.
EASE: Expression analysis systematic explorer
eIF: Eukaryotic translation initiation factor
GFP: Green fluorescent protein
GO: Gene ontology
GPCR: G-protein coupled receptor
GTP: Guanosine triphosphate
Hipi: Hyper-induction of peptide expression after infection
IRG: Infection response gene
KEGG: Kyoto encyclopedia of genes and genomes
Nipi: No induction of peptide after *Drechmeria* infection
Peni: Peptide expression no infection
ROS: Reactive oxygen species
TOF: Time of flight
TRG: Taxonomically-restricted gene
UPR: Unfolded protein response
UPR^mt^: Mitochondrial unfolded protein response
