# Supplementary material for: A quantitative genome-wide RNAi screen in C. elegans for antifungal innate immunity genes
Source: BMC Biol. 2016 Apr 29;14:35. doi: 10.1186/s12915-016-0256-3 (PMC4850687; doi:10.1186/s12915-016-0256-3)
Supplement: Additional file 4: Supplementary Methods. — Details of how Nipi clones where selected after the second round of screening. (PDF 60 kb) [file 12915_2016_256_MOESM4_ESM.pdf]

1. High-throughput RNAi screen in quadruplicate

The candidate RNAi clones that passed the first round of screening were cherry-picked to 18 new 96-well plates, each including 2 columns of negative controls (sjj_K04G11.4 and *sta-1* in column 1 and 11, respectively), together with different positive control clones, targeting known Nipi genes, such as *sta-2*, *rack-1* or *nipi-3*, in column 12. The screening procedure was exactly the same as for the first screen except that plates were screened 4 times each.

2. Screen analysis & Candidate selection

To analyse the results, we calculated the means of the 8 values obtained for the sjj_K04G11.4 negative control for each measured parameter on each plate and normalized the corresponding values for each well on that plate using these means.

Two methods of selection for Nipi clones were then performed. (1) 319 clones for which the Green/TOF value was under 0.8 in at least 3 tests, with Red/TOF values >0.4 were retained. (2) 309 clones were selected on the basis of the distribution of their normalized Green/TOF and Red/TOF values. Cutoffs were set to select clones with a low GFP level and a near-normal red expression. Clones were retained when at least 3 of their 4 values were below one of the 3 cutoffs (indicated by the green lines in the graph below).

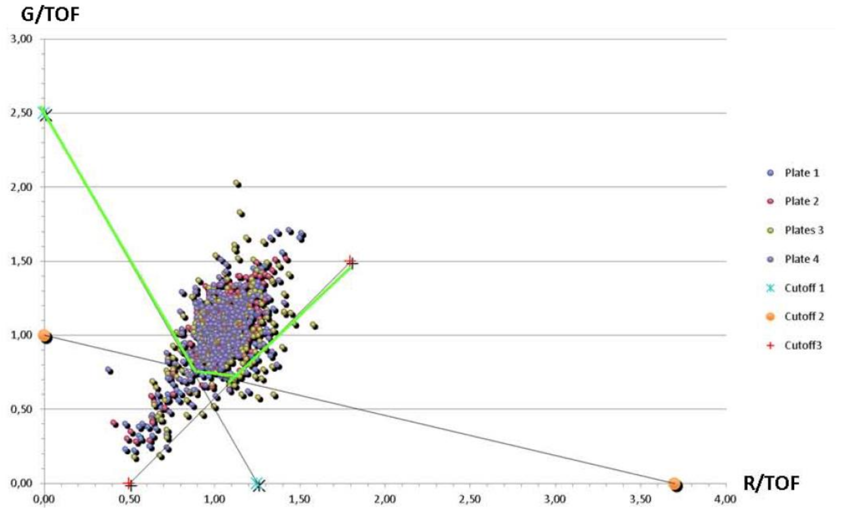

The lists were then combined to give a total of 360 Nipi clones, of which 269 had been selected by both methods. For more details see the publically available PhD thesis:

Squiban B: **Criblage par ARN interférence du génome complet de *C. elegans* pour l' identification de nouveaux gènes impliqués dans l' immunité innée.** Aix-Marseille Université; 2012.
